# What do international health electives and state examination scores have in common? – A cohort study to compare the results of written medical licensing examinations with the participation in international health electives during the final year of undergraduate medical education in Germany

**DOI:** 10.3205/zma001200

**Published:** 2018-11-30

**Authors:** Sylvère Störmann, Matthias W. Angstwurm

**Affiliations:** 1 Klinikum der Universität München, Medizinische Klinik und Poliklinik IV, München, Germany

**Keywords:** final year undergraduate medical education, international health electives, medical licensing examination

## Abstract

**Objective: **The final year of undergraduate medical education in Germany is called the practical year (PY), where emphasis is placed on developing practical skills requisite of soon-to-be physicians. Many students choose to spend part of this year abroad, yet little is known about their results on the medical licensing examinations. Is there a predisposition of high-performing students to go abroad as compared to lower performers? Are international health electives during the PY followed by higher scores in the final section of the German medical licensing examination (GMLE2)?

**Methods: **We conducted a retrospective cohort study among undergraduate medical students at the LMU Munich, who participated in the final section of the German medical licensing examination between autumn 2009 and spring 2011. Of the 1,731 eligible students, 554 (32%) participated in our study. We analyzed for statistical associations of international health electives with written test scores of both sections of the medical licensing examination as well as grades earned during medical school training. We then used multiple regression analysis to identify relevant predictors of GMLE2 scores.

**Results: **Approximately half of study participants pursued international health electives during the PY (51.1%). The number of students going abroad increased with the scores achieved on the first section of the medical licensing examination (GMLE1, p<0.001). Stratified by their GMLE1 scores students who pursued electives abroad during their PY achieved higher GMLE2 scores (p<0.001). The strongest predictor for GMLE2 scores were grades obtained during medical school training; age and study duration indicated lower scores; and those engaging in international health electives correlated with higher scores.

**Conclusions: **Students with higher GMLE1 scores go abroad during PY more often. Beyond that, students who pursue international health electives achieve higher GMLE2 scores than those who stay in Germany during PY. There is an unmet need for additional research to identify which factors make these students perform better and what motivates them to go abroad.

## Zusammenfassung

**Zielsetzung: **Das Praktische Jahr (PJ) im Medizinstudium nimmt eine Schlüsselrolle als letzte Praxisphase vor dem Berufseinstieg ein. Viele Studierende verbringen Teile davon im Ausland. Es ist wenig über deren schriftliche Leistungen in der Ärztlichen Prüfung bekannt, insbesondere im Hinblick darauf, ob speziell leistungsstärkere oder -schwächere Studierende Teile ihres PJ im Ausland verbringen und ob die Absolvierung von Teilen des PJ im Ausland mit besseren Prüfungsleistungen im anschließenden schriftlichen Teil der Ärztlichen Prüfung einhergeht.

**Methodik:** Wir führten eine retrospektive Kohortenstudie mit Studierenden der LMU München durch, die von Herbst 2009 bis Frühjahr 2011 den Zweiten Abschnitt der Ärztlichen Prüfung ableisteten. Unsere Stichprobe umfasst 554 von 1.731 Prüfungsteilnehmern (32%). Die schriftlichen Noten der beiden Abschnitte der Ärztlichen Prüfung sowie Noten der Leistungsnachweise nach § 27 der Approbationsordnung („Kliniks-Noten“) wurden auf Assoziationen zum Auslands-PJ statistisch untersucht. Weitere Einflussfaktoren auf die Note des Zweiten Abschnitts wurden anhand einer multiplen Regression geprüft.

**Ergebnisse: **Etwa die Hälfte der Teilnehmer (51,1%) verbrachte Teile des PJ im Ausland. Der Anteil von Studierenden, die im PJ ins Ausland gingen, war höher je besser die schriftliche Note im 1. Abschnitt der Ärztlichen Prüfung war (p<0,001). Stratifiziert nach dieser Note zeigten die Teilnehmer mit Auslands-PJ auch bessere schriftliche Noten im 2. Abschnitt der Ärztlichen Prüfung (p<0,001). Stärkster Prädiktor der Note im 2. Ärztlichen Prüfungsabschnitt waren die Kliniks-Noten. Höheres Alter und längere Studiendauer wiesen auf schlechtere und das Ableisten von PJ-Abschnitten im Ausland auf bessere Noten im 2. Ärztlichen Prüfungsabschnitt hin.

**Schlussfolgerung: **Studierende mit besseren schriftlichen Noten im 1. Ärztlichen Prüfungsabschnitt gehen häufiger im PJ ins Ausland. Darüber hinaus haben Studierende mit Auslands-PJ bessere schriftliche Prüfungsleistungen im 2. Ärztlichen Prüfungsabschnitt als die Studierenden, die ihr PJ vollständig in Deutschland ableisten. Welche Faktoren beeinflussen, warum diese Studierenden besser sind und was sie zum Auslands-PJ motiviert, bleibt noch zu klären.

## 1. Introduction

Undergraduate medical education in Germany includes two years of basic science training followed by the first section of the German medical licensing examination (GMLE1). Students then engage in four years of clinical training followed by the final section of the licensing examination (GMLE2). During the final year of undergraduate medical education in Germany students apply their knowledge of basic science and clinical theory in practice. This, this final year is called the Practical Year (PY) and helps students transition from the theory-based medical school approach into the professional experience of physicians. Provocatively, it can be regarded as the “last chance to render prospective doctors capable of performing their job” [1]. Regulatory specifications on state and federal levels ensure a high quality of the training during PY [2], but such regulations cannot be enforced let alone monitored abroad. The distinctive features of medical training in foreign countries encourage medical students to spend parts of their PY outside of Germany [3], [4]. Previous studies investigated international health electives in terms of cultural perception, consciousness of public health problems (especially in developing countries) and personal and professional development of participants [5], [6], [7].

There is a paucity of data on characteristics of students who choose to spend a portion of their medical training abroad. An analysis of the Center of Excellence of Medical Teaching Evaluation of Baden-Württemberg by Biller et al. showed better graduation grades and a shorter study duration among 208 medical students who went abroad during PY, as compared to 110 other students who stayed in Germany during PY [8]. This raises an important question: Do higher scores in previous exams correlate with a desire to pursue international health electives during PY?

Smilkstein and Culjat examined U.S. medical students who completed a 32-week primary care fellowship in Ghana and Nigeria. Evaluations before and after the fellowship covered 19 knowledge and skill variables in public health and in 18 areas students showed a significant increase in knowledge [9]. A previous study showed similar results in 1976 where USMLE Step 2 scores were compared between students who had passed an international fellowship program in Yugoslavia/Israel to the national average, as well as those who applied for the fellowship but were rejected. Participants of the fellowship scored significantly higher in the preventive medicine/public health portion of the examination, but not in other domains [10]. In contrast, Leeds et al. concluded that academic performance of participants versus nonparticipants in a surgical clerkship in Haiti were statistically insignificant [11]. However, the aforementioned studies describe structured, and partly specially funded programs with a pre-selection of participants. Medical students in Germany who spend part of their PY abroad organize their international health elective by themselves. The aforementioned study by Biller et al. showed higher GMLE2 scores in students who went abroad during PY, but these students were already considered higher performers as defined by à priori academic performance [8]. A study performed at the universities of Göttingen and Hamburg revealed improved performance on a test with 150 multiple choice question items when comparing the beginning to the end of the practical year [12]. This improvement was hypothesized to be the result of expanding knowledge by managing clinical cases. However, it remains unclear, whether going abroad during PY leads to higher GMLE2 scores afterwards.

The existing literature yields highly diverging numbers on the proportion and extent of international health electives during PY [4], [13], [14]. Therefore, we present data on which specialties and what countries students chose for their electives and analyze statistical associations with subsequent GMLE2 scores.

## 2. Methods

### 2.1. Study design and setting

We conducted a retrospective cohort study to evaluate written test scores and their statistical associations with international health electives during the practical year of undergraduate medical students at the LMU Munich. According to federal regulations at the time of the study (German Medical Licensure Act, Ärztliche Approbationsordnung, ÄAppO, cf. https://www.gesetze-im-internet.de/_appro_2002/BJNR240500002.html) all students had passed their GMLE1 after two years of basic science training, pursued three years of clinical lectures and training followed by the PY, a 48 week training in practice (subbdivided in 3 segments of 16 weeks each in internal medicine, surgery, and a third specialty freely chosen by the students). Finally, students passed their GMLE2 consisting of a written test with 320 multiple choice questions as well as an oral and practical examination. The validity and reliability of oral exams are deemed low as they are prone to grading errors and biases [15], [16]. In general students score higher in oral examinations [17], [18]. For this reason we only considered written test scores in this analysis. As mandated by federal regulations students passed several graded exams during clinical training (years three through five), which are referred to as “clinical grades” in this manuscript.

#### 2.2. Participants

All medical students were eligible to participate in a GMLE2-specific review course during their PY (LMU Staatsexamens-Repetitorium, LMU-StaR), a course designed by local staff and offered as of April 2009 until it was shut down due to changes in regulations [19]. The present investigation was conceived as a sub-project of LMU-StaR.

#### 2.3. Data acquisition

We assessed the following data: age, sex, study duration, clinical grades, dates and achieved grades of GMLE1 and GMLE2, and information on PY segments (dates, specialty, country, and continent of each segment). Sociodemographic data (age, sex, study duration) and examination-related data (clinical grades, dates and grades of the GMLE sections) were provided by the office of the dean of student affairs. We used the arithmetic mean of all clinical grades for calculations. Students had to register their PY segments with the dean of student affairs so that official information on the dates, specialty, and clinic were provided by the office. However, this information only detailed clinics in Germany whereas electives abroad were marked as “external.” We asked students to specify international health electives by providing country, city, and name of the clinic using an online form. We checked entries manually for completeness and accuracy. We corrected or completed missing or implausible entries by directly contacting students. Countries were categorized by continents. We constructed a dichotomous variable indicating whether a student had passed any PY segment abroad or not.

#### 2.4. Statistics

We used SPSS (IBM Corp., Armonk, NY, U.S.A.) for statistical analyses. We set the level of significance at α=0.05. We characterize the study cohort descriptively using means and standard deviations. Mean grades were calculated using the arithmetic mean and are given with their standard deviation. We performed a one-way analysis of variance to test for age differences of the participants of the different GMLE2 dates. We used Fisher’s exact test or the chi squared test, respectively, to assess for differences in the distribution of categorical data and present the results with Odd’s Ratio or Cramer-V respectively. We tested normal distribution of group data using the Kolmogorov Smirnov test. As all variables in question did not meet the assumption of normal distribution we used the Mann Whitney U test for comparison of group data. We evaluated differences in GMLE2 grades in respect to the destination countries of international health electives for each single country by comparing grades of students who went there with the grades of those who did not. We applied the Bonferroni correction to adjust for multiple testing and calculated effect sizes of group comparisons using Cohen’s d.

We performed a stepwise multiple regression analysis with GMLE2 grades as dependent variable (probability of F to enter p≤0.05). We excluded multicollinearity by checking for a Variance Inflation Factor (VIF) <10 as well as tolerance values >0.1 and ruled out auto-correlation of residuals with the Durbin-Watson statistic. Based on theoretical deliberation and where available previously described statistical associations we chose age, sex, study duration, clinical grades, and the number of weeks spent abroad during PY as predictors for the regression analysis [20], [21], [22]. We assessed R² as measure of variability explained by the predictors. We calculated Cohen’s f² to determine the effect size of our model. We used the regression model to compute a predicted value y of the GMLE2 grade [23], [24] and compared this to the actual GMLE2 grade using the Wilcoxon signed-rank test.

#### 2.5. Data protection and ethical approval

We reported the operational sequence, purpose, and intention of scientific interpretation of the data obtained through LMU-StaR to the local ethics committee, which deemed a formal ethical approval was not necessary. The study was conducted according to principles of the World Medical Association’s Declaration of Helsinki and Declaration of Geneva. All data were stored and analyzed after pseudonymization. Only one person knows the algorithm for the calculation of the pseudonym code which was stored in a file secured by two different cryptographic techniques using two completely different passwords. Participation in the LMU-StaR and the investigation at hand were voluntary and participants gave written consent to the analysis and anonymous publication of their data (performance scores, course of PY, sociodemographic data). We did not include any participant from LMU-StaR who did not give this consent.

## 3. Results

### 3.1. Study cohort

We considered all students eligible for our study who passed their GMLE2 between autumn 2009 and spring 2011 (n=1,731). A total of 554 students (32%) participated in our study. Of these, 67% were female and the mean age of our participants at the time of the GMLE2 was 27.9±4.4 years. The mean age of participants did not differ significantly over the course of the investigated examinations. Table 1 (Tab. 1) shows further details.

#### 3.2. PY segments abroad

Approximately half of our participants (48.9%; n=271) stayed in Germany for their PY. Accordingly, 51.1% of our study cohort went abroad during their PY (n=283). A majority of these 283 students (60.1%; n=170) spent up to 16 weeks abroad, which equates to roughly one third of the whole study cohort (30.7%). 92 students (32.5% of 283) spent 24 or 32 weeks abroad during their PY. A minority of students, 7.4% (n=21), spent most of their PY abroad, i.e. 40 to 48 weeks (equivalent to 3.8% of the study cohort). The proportion of time spent abroad during PY did not significantly differ between male and female students.

#### 3.3. PY abroad or not: Differences of GMLE1 grades and clinical grades

The clinical grades of the students in our sample differed significantly between students who went abroad during PY and those who stayed in Germany. The former had better grades (2.1±0.3) than the latter (2.2±0.3; U=28,853, p<0.001, Cohen’s d=0.377).

There was a significant relation between going abroad during PY and the GMLE1 grade (X²(3, N=554)=29,8, p<0,001, Cramer-V=0,232). The better the GMLE1 grades, the higher the proportion of students opting to go abroad during PY. Table 2 (Tab. 2) presents data on students going abroad during PY and the mean time spent abroad clustered by GMLE1 grades.

#### 3.4. PY abroad and GMLE2 grades

The Mann Whitney U test showed a significant difference in GMLE2 grades between study participants who went abroad during PY and those who did not (U=29,254, p<0.001, Cohen’s d=0.406). Students with PY abroad achieved a mean grade of 2.8±0.8 whereas those without international experience scored 3.1±0.7. This difference could still be observed when stratifying the study cohort according to GMLE1 grades (see Table 2 (Tab. 2)). Therefore, we performed a multiple regression analysis using the GMLE2 grades as a dependent variable and the variables age, study duration, clinical grades and weeks spent abroad during PY as predictors. Sex as a predictor variable did not achieve the significance threshold of 0.05 and was not included in the model. Our model R squared was 0.59 (F(4, 537)=195,957; p<0,001), and correspondingly showed a large effect size (Cohen’s f²=1,44). The Durbin-Watson statistic with a test value of 1.87 indicated no serial correlation of the residuals. The collinearity statistics of the predictors showed tolerance values between 0.85 and 0.972 and VIF values between 1.029 and 1.177 respectively, thus excluding multicollinearity. Table 3 (Tab. 3) presents the coefficients in detail. Clinical grades exerted the strongest influence with a standardized coefficient of 0.717. Age, weeks abroad and study duration played only a minor role. Higher age and longer study duration respectively displayed an unfavorable effect on GMLE2 grades, whereas time spent abroad during PY hinted at better grades. The following equation was constructed to calculate a prediction value y for GMLE2 grades: y=-1.324+1.664xclinical grades+0.014xage–0.004xweeks abroad during PY+0.021xstudy duration. The Wilcoxon signed-rank test showed no differences between the prediction value y and the factual GMLE2 grade obtained, neither for the entire cohort nor in sub-group analysis.

#### 3.5. Specialties and destination countries during PY abroad

The highest proportion of students spending a part of their PY abroad was during their surgical elective: 37.5% of participants (n=208) spent it completely (n=145) or partly (n=63) abroad. Almost one in four students of our cohort (23.5%, n=130) went abroad for their elective in internal medicine (completely: n=96; partly: n=34). During the elective where the students freely chose the specialty 17.9% (n=99) spent it abroad (completely: n=55). Comparing GMLE2 grades between students who went abroad and those who did not grouped by specialty showed no significant differences. 

The majority of PY segments spent abroad occurred in Europe: 222 students of our cohort (40.1%) spent 65.8% of all PY segments in Europe. In this context, Switzerland accounted for 42.6% of all PY segments abroad. Other popular countries were, in descending order, the United States, France, Australia, England, South Africa, Italy, Canada, and Spain. Table 4 (Tab. 4) presented the distribution of destination countries categorized by continents. GMLE2 grades did not differ significantly stratified by destination country.

## 4. Discussion

In our retrospective cohort study we observed a better performance on the written portion of GMLE2 amongst study participants who spent their PY partly or completely abroad as compared to those who spent their whole PY in Germany. At closer inspection à priori differences of academic achievement were noticeable as students in our cohort who went abroad also had better GMLE1 and clinical grades. The worse the GMLE1 grade the smaller the proportion of students opting for an international health elective during PY (see Table 2 (Tab. 2)). A previous study from Baden-Württemberg indicated similar results [8]. As shown in a systematic review academic achievement serves as a good predictor of future performance in medical practice [25].

Beyond that we showed that even after stratification by GMLE1 grades our students with PY abroad achieved better GMLE2 grades than those without PY abroad. Regression analyses showed a weak, but statistically significant association between PY abroad and GMLE2 grades independent of other factors such as clinical grades, age, and study duration. However, this does not directly infer an immediate positive effect of spending one’s PY abroad on GMLE2 grades. As grounds for discussion additional factors not captured in this study could influence the course of studies in terms of higher academic achievement during undergraduate medical education and lead to a disposition to go abroad. In our study we characterize the students solely by demographic data and their exam results. We did not assess language skills or personal connections to the destination countries (e.g. students with a migration background). It is unclear to what extent study participants differed in terms of character, personality, bonding patterns or anxiety traits (especially regarding test anxiety), as such traits could influence study course, readiness to pursue experiences abroad and academic performance [26], [27]. Furthermore, we did not assess external factors such as income/fortune and parental/peer pressure [28], [29] which could have influenced results.

Other limitations of this study encompass the bias induced by the cohort selection which was from a single university and included only 32% of all eligible students. The study design allows only for the detection of statistical associations but not causality. We recruited participants via LMU-StaR which may have introduced another selection bias. However, an independent analysis showed no difference of LMU-StaR participants compared to all other PY students in terms of age, sex, graduation grade and GMLE1 grades [30]. Beyond that the presented data originates from a cohort whose curriculum differed in some aspects from the current curriculum. Therefore, findings from this analysis might not apply to today’s medical students. Finally, to respect validity we focused exclusively on grades from the written parts of the medical licensing examinations [31] and did not consider grades from the oral examinations which may have revealed further associations.

In 2012 Holmes assembled central objectives of international health electives in a survey among medical students:

1. to observe the practice and organization of health care in another country;
2. improve medical/surgical skills;
3. improve language skills;
4. learn about another culture; and
5. deepen knowledge of infectious disease [32].

Fundamentally, this conforms to a more comprehensive literature review by Cherniak and colleagues, albeit primarily focused on structured programs [33], and a personal opinion article authored by medical students [3]. 

During international health electives students may gain practical experience by exerting tasks they are not qualified to do and thus increasing practical skills on the job. This idea was illustrated by Radstone in a small survey on the Solomon Islands where all healthcare staff at one hospital acknowledged the belief that medical students should be allowed to diagnose, treat, and prescribe without direct supervision from a qualified doctor [34]. Therefore, going abroad may provide students with the opportunity to obtain practical hands-on experience they do not receive in their home country [35], [36]. Hence, it is conceivable that in our sample an intrinsic motivation for self-actualization and practical experience played an important role, which might partly explain the observed associations.

The top-ranking destination country in our study was Switzerland, which might be due to it being a German-speaking country [37], its proximity, and payment of salary (which at the time of the study was not permitted in Germany) [13]. Secondary motivations such as reimbursement and recreational offerings of destination countries are important reasons why international exchange is not only marked by the pursuit of idealistic motives [38]. Furthermore, students often do not prepare for international health electives [39]. On top of this, the planning of an international health elective is laborious, costly, occasionally risky and may be hindered by private or occupational liabilities [7]. Another barrier to experiences abroad is the lack of structured offerings and educational objectives which may impede academic development [40]. These aspects and their impact on academic and clinical performance should be highlighted in further investigations. At least in our study going abroad during PY did not negatively influence performance on the second section of the medical licensing examination.

## 5. Conclusions

Students with better GMLE1 grades choose to go abroad during PY more often. In additional, students going abroad during PY achieve significantly better GMLE2 grades than those who remained in their home country, even when stratified by baseline GMLE1 grades. It remains unclear and cannot be elucidated by this investigation which individual factors are relevant for academically successful students who opt for international experiences. Further studies are necessary to identify these factors and provide possible starting points for targeted support.

## Competing interests

The authors declare that they have no competing interests. 

## Figures and Tables

**Table 1:**
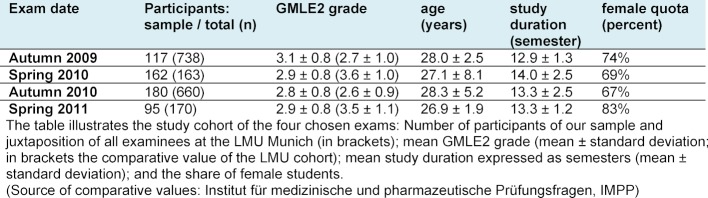
Description of the study sample

**Table 2:**
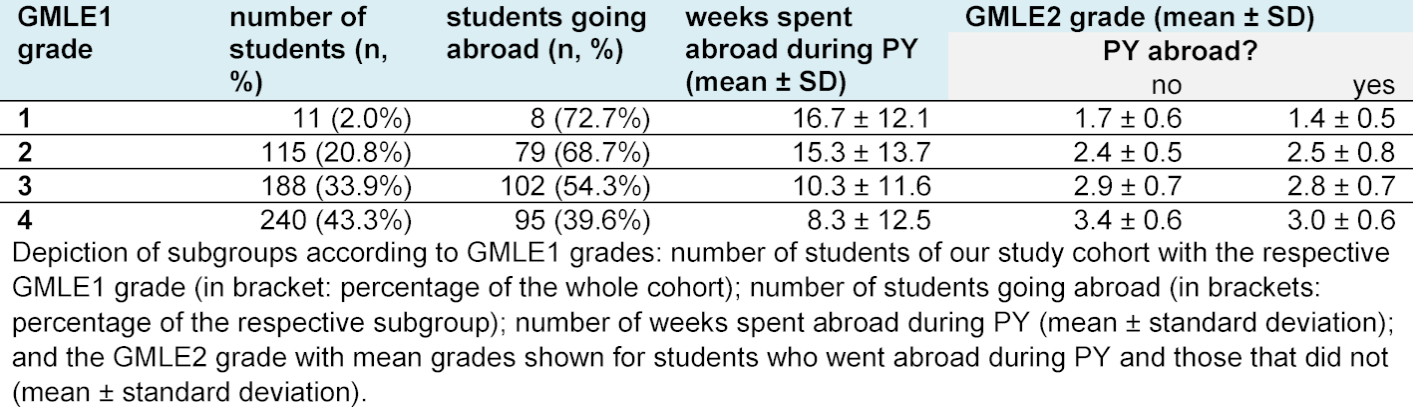
PY segments abroad and GMLE2 grades

**Table 3:**
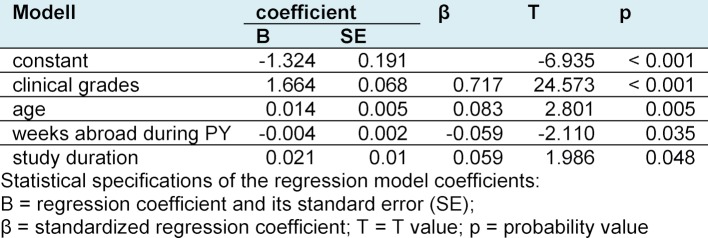
Coefficients of the regression model

**Table 4:**
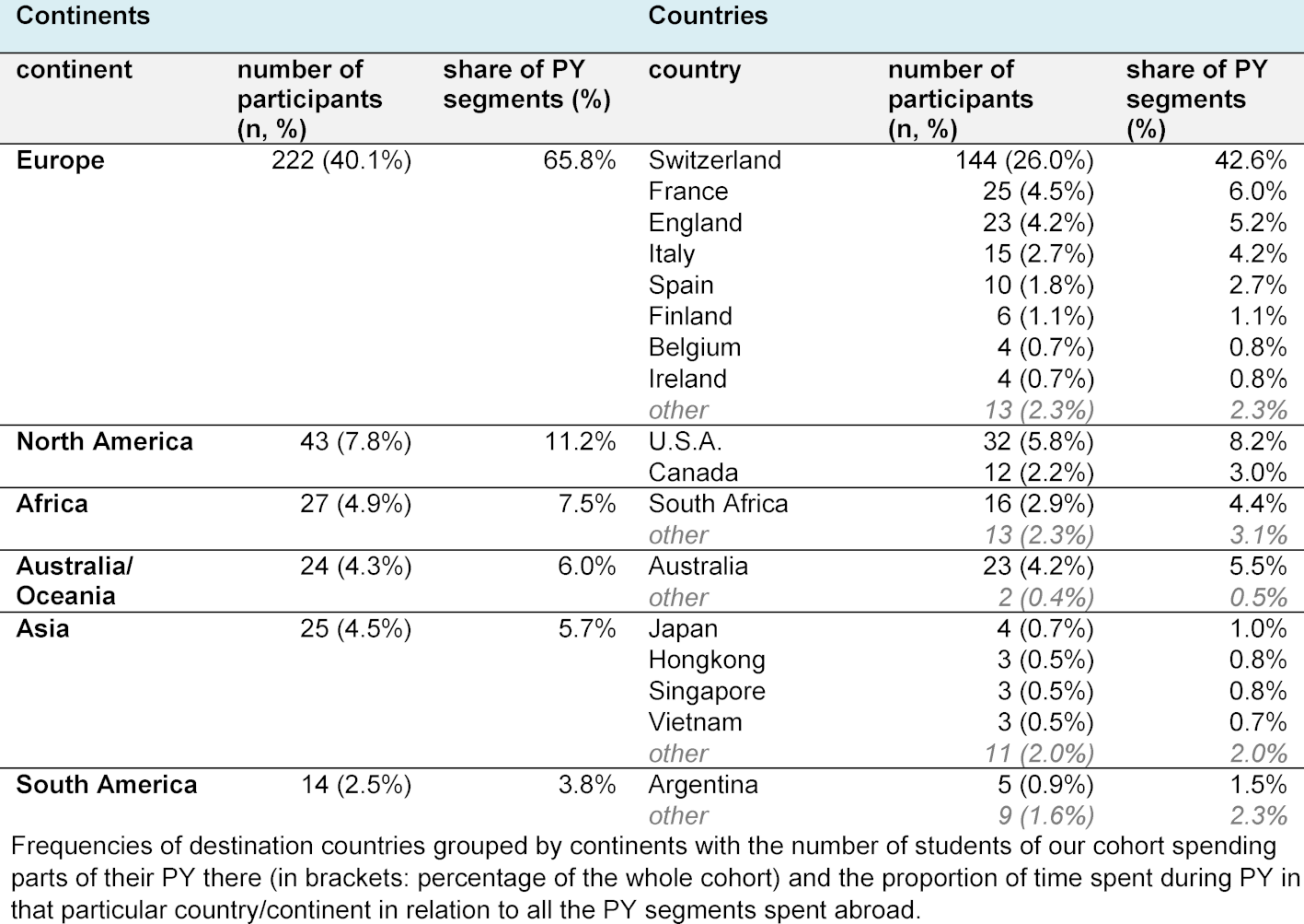
Destination countries of PY segments spent abroad
